# Associations between family history and coronary artery calcium and coronary heart disease in British Europeans and South Asians^☆^

**DOI:** 10.1016/j.ijcard.2019.07.101

**Published:** 2020-02-01

**Authors:** Jingyi Wang, Therese Tillin, Alun D. Hughes, Nish Chaturvedi

**Affiliations:** MRC Unit for Lifelong Health and Ageing, Institute of Cardiovascular Science, University College London, Floor 5, 1 – 19 Torrington Place, London WC1E 7HB, United Kingdom

**Keywords:** Family history, Coronary heart disease, Coronary artery calcium, South Asian, European

## Abstract

**Background:**

The role of family history in determining excess risk of coronary heart disease (CHD) in South Asians compared with Europeans is unclear. We hypothesized that family history would be more strongly associated with CHD in South Asians.

**Methods:**

We performed cross-sectional analyses of 20-year follow-up data from SABRE (Southall And Brent Revisited) population-based study. Initial recruitment (1988–1991) was by random selection from general practitioners' lists in north-west London. 974 Europeans and 734 South Asians completed follow-up questionnaire (2008–2011) and had complete CHD and family history data. 831 participants without cardiovascular disease had complete coronary artery calcium (CAC) data.

**Results:**

South Asians with family history of CHD were more likely to have diagnosed CHD (odds ratio 1.71 [95% CI 1.21, 2.42]; *p* = 0.002) and more previous CHD events (regression coefficient 0.44 [0.16, 0.72]; p = 0.002) than those without family history, independent of biological and sociodemographic risk factors. Family history associations with diagnosed CHD/number of events were weaker in Europeans (odds ratio 1.11 [0.78, 1.57]; *p* = 0.562/regression coefficient 0.02 [−0.25, 0.30]; *p* = 0.878), largely explained by biological risk factors. South Asians with family history had modestly increased CAC burden compared with Europeans.

**Conclusions:**

There were markedly stronger associations between family history and clinical CHD in South Asians, and a similar trend for subclinical CHD. Early preventive and therapeutic interventions are particularly important in South Asians with a family history of CHD.

## Highlights

• Family history of CHD is greatly associated with clinical CHD in South Asians.
• The associations are stronger in British South Asians than Europeans.
• There is a similar trend for subclinical CHD.
• The associations in South Asians are unexplained by conventional risk factors.

## 1 Introduction

Coronary heart disease (CHD) is the leading cause of global mortality and morbidity [1] with a particularly high burden in South Asians [2]. Approximately 3 million South Asian migrants live in the UK [3] and experience both earlier onset and three- to four-fold increased risk of CHD, unexplained by conventional risk factors [4,5].

The aims of this study were to compare cross-sectional associations between family history of CHD and 1) subclinical CHD, 2) diagnosed CHD, 3) number of previous CHD events in a group of British South Asians and Europeans. We hypothesized that family history would increase CHD risk in South Asians to a greater extent than in Europeans. We also aimed to examine the extent to which the family history effect, capturing genetic and early life influences on CHD risk, would be explained by sociodemographic or biological risk factors in each ethnic group.

## 2 Methods

### 2.1 Data collection

We used data from the 20-year follow-up of the SABRE (Southall And Brent REvisited) cohort [6]. At baseline (1988–1991) 2346 Europeans and 1710 South Asians (all first generation migrants) were recruited from general practitioners' lists in north-west London. Ethnicity was confirmed based on self-description and parental origins. At the 20-year follow-up (2008–2011) 1757 (75%) Europeans and 1349 (79%) South Asians were alive. Of survivors, 974 Europeans (55%) and 734 South Asians (54%) completed a family history, health and lifestyle questionnaire (*n* = 1708). 684 (39%) Europeans and 522 (39%) South Asians attended for clinical follow-up, including coronary artery calcium (CAC) measured in Agatston units (AU) using computerised tomography [6]. Subclinical CHD was categorised into four levels of CAC score (0 AU, 1–100 AU, >100–400 AU, >400 AU) [7] and excluded those with known cardiovascular disease (CVD). Diagnosed CHD/number of CHD events were identified from hospital admissions or primary care record review [5]. Family history of CHD was identified from self-reported diagnosis of angina or heart attack in parents or siblings at any age. St Mary's Hospital Research Ethics Committee (07/H0712/109) approved the study protocol, and all participants provided written informed consent.

### 2.2 Statistical analysis

All 1708 questionnaire responders had complete CHD and family history data, while smoking, years of education and early life disadvantage had missing values ranging from 11 (0.6%) to 114 (6.7%). 831 clinic attenders without known CVD had complete CAC data. Missing data were imputed using multiple imputation by chained equations on ethnic subsamples. The distribution of CAC score is right-skewed with excessive zeros (histogram and Skewness/Kurtosis tests). No transformation yielded normal distribution, so CAC score was classified into four categories. Generalized logistic regression (partial proportional odds) [8] provided odds ratios (ORs) and 95% confidence intervals (CIs) for associations between family history and CAC categories as the proportional odds assumption was not met. Logistic regression was used for diagnosed CHD (binary variable); ORs (95% CI) were reported. Number of CHD events is an over-dispersed count variable, therefore negative binomial regression was used and regression coefficients (95% CI) were reported. Interactions between family history and ethnicity were tested for each outcome. Model 1 adjusted for age and sex, model 2 added sociodemographic factors (smoking, years of education, early life circumstances), and model 3 added biological risk factors (diabetes, lipid lowering medications, antihypertensive medications). Model 4 included both sociodemographic and biological risk factors. Sensitivity analyses included complete case analyses and additional adjustment for HbA1_c_, cholesterol:HDL ratio, waist-to-hip ratio, systolic blood pressure and hypertension in clinic attenders (*n* = 1206). All analyses were conducted in Stata version 14.2.

## 3 Results

The mean (SD) age of the sample was 70.1 (6.4) years, and by design the majority (79.5%) were male. Family history of CHD was reported by 490 (50.3%) Europeans and 309 (42.1%) South Asians. South Asians experienced more CHD and more previous CHD events. Total CAC levels and CAC levels within each of the main coronary arteries were similar in both ethnic groups (Table 1).

**Table 1:** Characteristics of the study population by family history status across ethnic groups (*n* = 1708). Observed data (no imputations).

Characteristics	European			South Asian			*p* value†
	With family history of CHD (*n* = 490)	No family history of CHD (*n* = 484)	p value⁎	With family history of CHD (*n* = 309)	No family history of CHD (*n* = 425)	p value⁎	
Age, yr	70.4 ± 6.3	71.1 ± 6.6	0.117	68.1 ± 5.6	70.2 ± 6.3	<0.001	<0.001
Female	126 (25.7)	102 (21.1)	0.087	51 (16.5)	72 (16.9)	0.876	0.002
Smoking (ever)	289 (59.2)	326 (67.6)	0.007	83 (27.1)	79 (18.8)	0.007	<0.001
Education, yr	11.1 ± 2.7	10.9 ± 2.8	0.259	12.8 ± 3.5	12.4 ± 3.9	0.182	<0.001
Early life circumstances^a^	−0.2 ± 0.6	−0.2 ± 0.6	0.731	0.1 ± 0.6	0.2 ± 0.7	0.030	<0.001
Diabetes	94 (19.2)	95 (19.6)	0.861	138 (44.7)	189 (44.5)	0.959	<0.001
Use of lipid lowering medications	256 (52.2)	218 (45.0)	0.025	218 (70.6)	271 (63.8)	0.054	<0.001
Use of antihypertensive medications	284 (58.0)	261 (53.9)	0.205	242 (78.3)	312 (73.4)	0.127	<0.001
Number of CHD events (categories)							
No CHD	375 (76.5)	383 (79.1)	0.807	178 (57.6)	292 (68.7)	0.005	<0.001
1 CHD event	68 (13.9)	59 (12.2)		54 (17.5)	66 (15.5)		
2 CHD events	24 (4.9)	21 (4.3)		30 (9.7)	20 (4.7)		
≥3 CHD events	23 (4.7)	21 (4.3)		47 (15.2)	47 (11.1)		
Coronary artery calcification (*n* = 995)	(*n* = 300)	(*n* = 301)		(*n* = 165)	(*n* = 229)		
0 AU	62 (20.7)	65 (21.6)	0.667	30 (18.2)	43 (18.8)	0.459	0.858
1–100 AU	81 (27.0)	93 (30.9)		49 (29.7)	84 (36.7)		
>100–400 AU	79 (26.3)	73 (24.3)		41 (24.9)	50 (21.8)		
>400 AU	78 (26.0)	70 (23.3)		45 (27.3)	52 (22.7)		

Values are mean ± SD or No. (%). CHD, coronary heart disease; AU, Agatston Units.

⁎ p value comparing those with and without a family history of CHD within each ethnic group.

† *p* value comparing only those with a positive family history of CHD by ethnicity.

a A global measure of early life circumstances was calculated as a composite z score of father manual occupation, people/room at age 12 and household amenities at age 12, with higher scores indicating poorer early life circumstances.

**Fig. 1:**
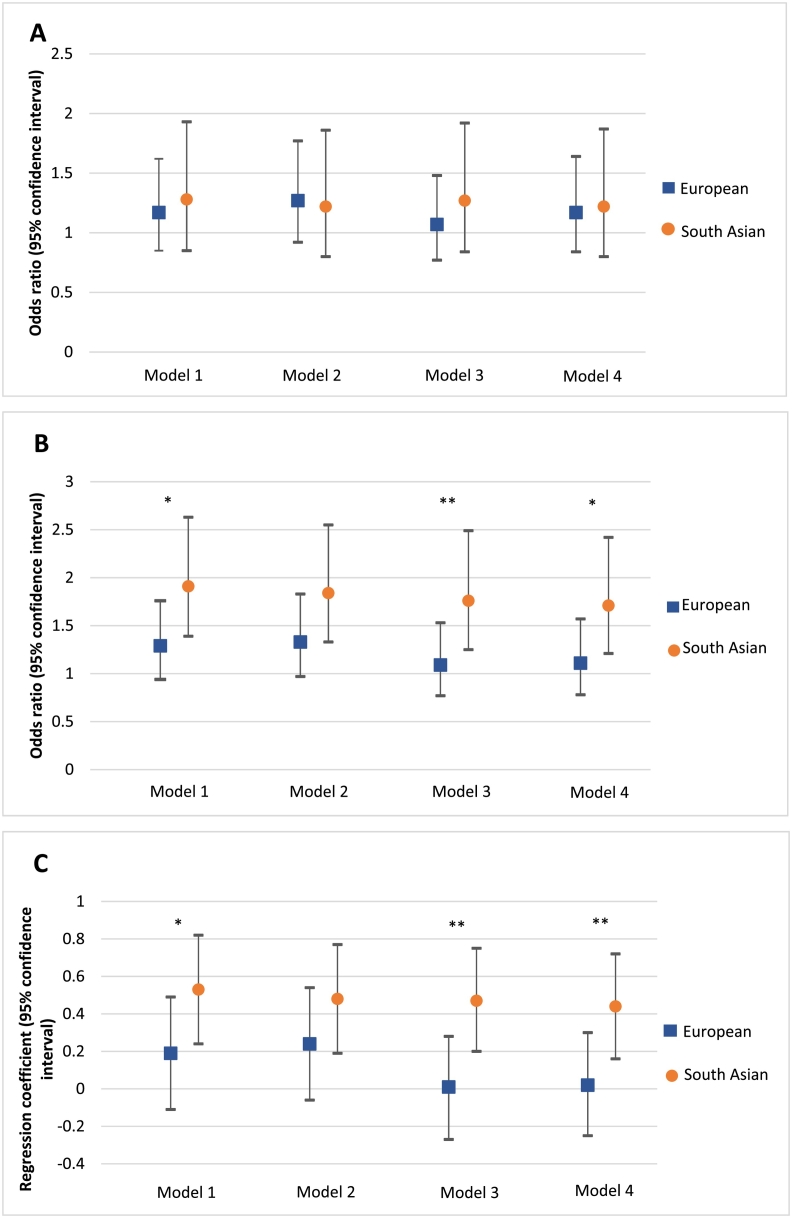
Associations between family history of CHD and subclinical (CAC) and clinical (diagnosed CHD) outcomes stratified by ethnicity. 1A: The effect sizes in the association between family history of CHD and CAC categories (0 AU; 1–100 AU; >100–400 AU; >400 AU) in responders without CVD (*N* = 831), stratified by ethnicity in generalized logistic regression (partial proportional odds). Odds ratios indicate the likelihood of being in a worse CAC category for people with positive family history of CHD. 1B: The effect sizes in the association between family history of CHD and diagnosed CHD in all questionnaire responders (*N* = 1708), stratified by ethnicity in logistic regression. 1C: The effect sizes in the association between family history of CHD and number of CHD events in all questionnaire responders (N = 1708), stratified by ethnicity in negative binomial regression. Model 1 was adjusted for age and sex. Model 2 was additionally adjusted for smoking, years of education and early life circumstances. Model 3 was adjusted for model 1 plus diabetes, lipid lowering medications and antihypertensive medications. Model 4 included adjustment for model 2 and model 3. *p < 0.1 for interaction family history of CHD – ethnicity, **p < 0.05 for interaction family history of CHD – ethnicity.

Among participants without diagnosed CVD, South Asians with family history were 1.28 times (95% CI 0.85, 1.93; *p* = 0.243) more likely to be in a worse CAC category compared to those without family history (model 1). The association was weaker in Europeans (OR 1.17 [0.85, 1.62]; *p* = 0.331) and largely explained by biological risk factors (OR 1.07 [0.77, 1.48]; *p* = 0.679), especially lipid lowering and antihypertensive medications (model 3). In fully adjusted model 4, the OR was 1.17 (0.84, 1.64; *p* = 0.357). In South Asians, however, the association between family history and CAC categories was unchanged after adjustment for sociodemographic or biological risk factors (fully adjusted OR 1.22 [0.80, 1.87]; *p* = 0.352) (Fig. 1A).

Among questionnaire responders, South Asians with family history of CHD were more likely to have diagnosed CHD (OR 1.91 [1.39, 2.63]; *p* < 0.001) and more CHD events (regression coefficient 0.53 [0.24, 0.82]; p < 0.001) than those without family history (model 1). These associations were hardly explained by sociodemographic or biological risk factors (OR 1.71 [1.21, 2.42], *p* = 0.002; regression coefficient 0.44 [0.16, 0.72], p = 0.002 in model 4). However, the associations in Europeans were weaker and largely explained by biological risk factors (OR 1.11 [0.78, 1.57], *p* = 0.562; regression coefficient 0.02 [−0.25, 0.30], *p* = 0.878 in model 4). Ethnicity x family history interactions were strong after adjustment for biological risk factors (OR 1.63 [1.01, 2.63], *p* = 0.046; regression coefficient 0.47 [0.08, 0.86], *p* = 0.019) (Fig. 1B and C).

Sensitivity analyses, additionally adjusting for HbA1c, cholesterol:HDL ratio, waist-to-hip ratio, systolic blood pressure and hypertension, did not alter the results. The associations between family history and the three outcomes in Europeans were largely explained by biological risk factors, especially lipid lowering medications and hypertension, while unchanged in South Asians. Results based on complete case analysis were consistent with primary analyses.

## 4 Discussion

Family history of CHD was more strongly associated with diagnosed CHD and with more previous CHD events in British South Asians than Europeans. There was also a trend towards increased association between family history and subclinical disease (CAC burden) in South Asians.

In Europeans adjustment for biological risk factors diminished family history effect, mostly due to adjustment for lipid lowering and antihypertensive medications-presumably partially mediating this effect. In South Asians, family history associations were independent of biological and sociodemographic risk factors.

Few studies have compared associations between family history and CHD outcomes in South Asians and Europeans. Associations between family history and CAC were stronger in US South Asians compared with non-Hispanic Whites in the MESA/MASALA studies [9], although the study groups were larger and younger and South Asians were of higher socioeconomic position than in SABRE. In contrast, INTERHEART, a case-control study, reported equivalent associations across world regions between CHD and parental history, with minimal attenuation on risk factor adjustment [10]. However, as all cases were recruited with acute myocardial infarction within 24 h of symptom onset, there may be differential recall bias between cases and controls.

Limitations of our study include the potential for recall bias of family history. However, in the subclinical sample, overestimation of family history is unlikely. Moreover, as South Asians in our study were first-generation migrants and might not have frequent contact with their family, reporting of positive family history was probably underestimated. Another limitation relates to our participants' relatively old age, missing selective early mortality in those with a strong family history of disease. However, this is likely to affect South Asians more than Europeans, given the earlier age at disease onset and potentially greater impact of family history. Consequently, a persistent excess risk of family history at an older age in South Asians is even more remarkable. In addition, the use of categorical variable of CAC due to the right-skewed distribution of continuous CAC score may lose some information in the analyses. However, the classification of CAC score has been published before [7].

Conventional risk factors have not completely explained the excess CHD burden in South Asians [5]. A “thrifty gene” theory [11] may explain a shared predisposition to diabetes, obesity and CHD risk. Although these genes may confer a survival advantage when food is scarce, they may predispose individuals to cardiometabolic disorders in an obesogenic environment [2]. However, genome-wide association studies have not identified differences in allele frequencies or effect sizes in known loci to explain the increased CHD risk in South Asians, despite adverse cardiometabolic profiles being found in second and third generation of British South Asian children [12,13]. This may suggest that pre-natal and early life exposures play a more important role in the excess CHD risk in South Asian immigrants, or that interactions between genetic and environmental exposures differ by ethnicity. It is also possible that differences in proinflammatory state, endothelial dysfunction or oxidative stress contribute to ethnic disparities [14,15].

## 5 Conclusions

Our findings of markedly stronger associations of family history with clinical CHD in South Asians, and a trend with subclinical CHD, unexplained by conventional biological and sociodemographic factors, have important research and clinical implications. We suggest that other factors contributing to excess risk of CHD in South Asians should be explored and that family history should be a strong incentive to instigate early preventive and therapeutic measures.

## Conflicts of interest

None.

## References

[1] Roth G.A., Collaborotors G.B.D.C.D., Abate D., Abate K.H., Abay S.M., Abbafati C. (2018). Global, regional, and national age-sex-specific mortality for 282 causes of death in 195 countries and territories, 1980-2017: a systematic analysis for the Global Burden of Disease Study 2017. Lancet..

[2] Fernando E., Razak F., Lear S.A., Anand S.S. (2015). Cardiovascular disease in South Asian migrants. Can. J. Cardiol..

[3] 2011 Census: Key Statistics and Quick Statistics for Local Authorities in the United Kingdom. Defining characteristics of the UK population on the topics of population, ethnic group and country of birth, health and housing and accommodation. Office for National Statistics; 2013. https://www.ons.gov.uk/peoplepopulationandcommunity/populationandmigration/populationestimates/bulletins/keystatisticsandquickstatisticsforlocalauthoritiesintheunitedkingdom/2013-10-11. Accessed 15th January, 2019.

[4] Barnett A.H., Dixon A.N., Bellary S., Hanif M.W., O'Hare J.P., Raymond N.T. (2006). Type 2 diabetes and cardiovascular risk in the UK south Asian community. Diabetologia..

[5] Tillin T., Hughes A.D., Mayet J., Whincup P., Sattar N., Forouhi N.G. (2013). The relationship between metabolic risk factors and incident cardiovascular disease in Europeans, South Asians, and African Caribbeans SABRE (Southall and Brent Revisited)-a prospective population-based study. J. Am. Coll. Cardiol..

[6] Tillin T., Forouhi N.G., McKeigue P.M., Chaturvedi N., Grp S.S. (2012). Southall And Brent REvisited: Cohort profile of SABRE, a UK population-based comparison of cardiovascular disease and diabetes in people of European, Indian Asian and African Caribbean origins. Int. J. Epidemiol..

[7] Patel J., Al Rifai M., Blaha M.J., Budoff M.J., Post W.S., Polak J.F. (2015). Coronary artery calcium improves risk assessment in adults with a family history of premature coronary heart disease. Results from multiethnic study of atherosclerosis. Circ. Cardiovasc. Imaging.

[8] Williams R. (2006). Generalized ordered logit/partial proportional odds models for ordinal dependent variables. Stata J..

[9] Patel J., Al Rifai M., Cainzos-Achirica M., Kandula N.R., Kanaya A.M., Khera A. (2017). Family history of CHD is associated with severe CAC in South Asians. JACC Cardiovasc. Imaging.

[10] Chow C.K., Islam S., Bautista L., Rumboldt Z., Yusufali A., Xie C.C. (2011). Parental history and myocardial infarction risk across the world. The INTERHEART study. J. Am. Coll. Cardiol..

[11] Neel J.V. (1962). Diabetes mellitus: a “thrifty” genotype rendered detrimental by “progress”?. Am. J. Hum. Genet..

[12] Whincup P.H., Gilg J.A., Papacosta O., Seymour C., Miller G.J., Alberti K. (2002). Early evidence of ethnic differences in cardiovascular risk: cross sectional comparison of British South Asian and white children. Br. Med. J..

[13] Stanfield K.M., Wells J.C., Fewtrell M.S., Frost C., Leon D.A. (2012). Differences in body composition between infants of South Asian and European ancestry: the London Mother and Baby Study. Int. J. Epidemiol..

[14] Volgman A.S., Palaniappan L.S., Aggarwal N.T., Gupta M., Khandelwal A., Krishnan A.V. (2018). Atherosclerotic cardiovascular disease in South Asians in the United States: epidemiology, risk factors, and treatments: a scientific statement from the American Heart Association. Circulation.

[15] Brady E.M., Webb D.R., Morris D.H., Khunti K., Talbot D.S.C., Sattar N. (2012). Investigating endothelial activation and oxidative stress in relation to glycaemic control in a multiethnic population. Exp. Diabetes Res..

